# Shiga Toxin‒Producing *Escherichia coli* Diagnoses from Health Practitioners, Queensland, Australia

**DOI:** 10.3201/eid3001.231202

**Published:** 2024-01

**Authors:** Ashish C. Shrestha, Russell Stafford, Robert Bell, Amy V. Jennison, Rikki M.A. Graham, Emma Field, Stephen B. Lambert

**Affiliations:** Queensland Health, Brisbane, Queensland, Australia (A.C. Shrestha, R. Stafford, R. Bell, A.V. Jennison, R.M.A. Graham, S.B. Lambert);; Australian National University, Canberra, Australian Capital Territory, Australia (A.C. Shrestha, E. Field)

**Keywords:** Shiga toxin‒producing Escherichia coli, bacteria, STEC, hemolytic uremic syndrome, HUS, *stx*, bloody diarrhea, diagnoses, alternative health practitioners, diagnostic laboratories, Queensland, Australia

## Abstract

In Queensland, Australia, 31 of 96 Shiga toxin‒producing *Escherichia coli* cases during 2020–2022 were reported by a specialty pathology laboratory servicing alternative health practitioners. Those new cases were more likely to be asymptomatic or paucisymptomatic, prompting a review of the standard public health response.

Shiga toxin‒producing *Escherichia coli* (STEC) cause gastrointestinal illness and can result in hemolytic uremic syndrome (HUS) (*1*). Asymptomatic STEC infections can occur and might remain undetected (*2*,*3*), making the population incidence of STEC higher than reported through routine surveillance. In Australia, laboratory-confirmed STEC, based on isolation by culture or detection of *stx* gene(s) by nucleic acid testing of feces, is a nationally notifiable condition (*4*). In 2022, the national notification rate was 3.2 cases/100,000 population/year in Australia and 0.6 cases/100,000 population/year in Queensland (*5*).

The frequency of asymptomatic STEC cases increased in Queensland from 2% in 2018‒2019 to 29% in 2022. We reviewed the reports for 2020‒2022 and found that an increasing number of STEC cases had been reported from a specialty pathology laboratory (SPL) in the state of Victoria that services healthcare providers, including alternative health practitioners (naturopaths and nutritionists).

We undertook further analysis to clarify the reason for increasing case numbers. This analysis involved descriptive analysis of STEC case data extracted from the Queensland Health Notifiable Conditions System database and case report forms for January 2020‒December 2022. Ethics approval for this study was obtained from the Australian National University (protocol 2017/909).

SPL diagnosed STEC by performing multiplex PCR for enteric pathogens on fecal samples from patients. STEC confirmation and characterization of culture-positive isolates were performed subsequently by the Microbiology Diagnostic Unit Public Health Laboratory (Doherty Institute, University of Melbourne, Melbourne, Victoria, Australia). Other STEC cases referred to in this study were tested by pathology laboratories or the Queensland STEC reference laboratory (Public Health Microbiology, Forensic and Scientific Services, Queensland Health) by using PCR or culture. Additional confirmatory testing (culture, PCR, serotyping, genomic analysis) were performed by the reference laboratory.

STEC was reported from an SPL to Queensland Health on March 13, 2020. During 2020–2022, a total of 96 STEC cases were reported, 31 (32%) from the SPL and 65 (68%) from other pathology laboratories that provide services for medical practitioners only (Table; Figure). SPL-reported case-patients were more commonly female (81%) compared with other pathology laboratories (43%) (Table). Of the SPL-diagnosed cases, 85% (23/27) had stool testing requested by alternative health practitioners, naturopath (n = 19) or nutritionist (n = 4); 15% (4/27) were requested by medical practitioners, and the request source was unknown for 4 other cases. Of the case-patients diagnosed by pathology laboratories other than SPL, 92% (60/65) consulted medical practitioners, 6% (4/65) were identified during public health follow-up as a close contact of a previously reported case-patient, and 2% (1/65) were diagnosed after fecal donor screening.

**Table:** Characteristics of 31 STEC cases diagnosed by the specialty pathology laboratory and other pathology laboratories, Queensland, Australia, 2020–2022*

Characteristics	Specialty pathology laboratory			Other pathology laboratories		p value
	Value	% (95% CI)		Value	% (95% CI)	
Sex, no. (%)						
M	6	19 (9–38)		37/65	57 (44–69)	0.001
F	25	81 (62–91)		28/65	43 (31–55)	
Median age, y (range)	35 (1–65)			31 (<1–90)		
Clinical manifestation						
Symptomatic†	16/29 (55)	36–73		56/64	88 (77–94)	0.001
Bloody diarrhea	1/29 (3)	0–22		37/64	58 (45–69)	<0.001
HUS (% of all cases)	0	0		9/64	14 (7–25)	0.024
Hospitalized	0	0		27/62	4 (32–56)	<0.001
Household contacts‡	0	0		6/65	9 (4–19)	0.174
Laboratory culture positive	20/30	67 (47–83)		27/65	42 (29–54)	0.023
*stx* genes						
* stx1* positive, *stx2* negative	6/26	23 (9–44)		14/65	22 (12–33)	0.873
* stx2* positive, *stx1* negative	9/26	35 (17–56)		33/65	51 (38–63)	0.059
* stx1* positive,* stx2* positive	11/26	42 (23–63)		18/65	28 (17–40)	0.176
*eaeA *(intimin) positive	1/5	20 (1–72)		20/39	51 (35–68)	0.348
*ehxA *(enterohemolysin) positive	4/4	100 (40–100)		26/36	72 (55–86)	0.558
Serotypes known to cause severe disease						
O111	1/20	5 (1–25)		2/28	7 (1–24)	0.762
O157	0	0		6/28	21 (8–41)	0.034
O26	0	0		2/28	7 (1–24)	0.504
O145	0	0		2/28	7 (1–24)	0.504

*Values are no. cases or no. positive/no. tested except as indicated. Denominators reflect total cases where the relevant field was completed. HUS, hemolytic uremic syndrome; STEC, Shiga toxin‒producing *Escherichia coli.*†Gastrointestinal symptoms. ‡Includes contacts of a case and are cases of the study population.

**Figure:**
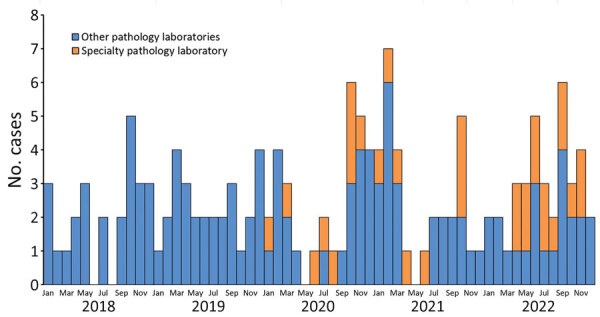
Shiga toxin‒producing *Escherichia coli* cases by month and year of episode date (earliest of specimen collection/onset dates) and reporting laboratories, Queensland, Australia, 2018–2022.

More case-patients given a diagnosis by other pathology laboratories were symptomatic, experienced bloody diarrhea, and were hospitalized than were SPL-diagnosed case-patients (Table). HUS was reported in case-patients given a diagnosis by other pathology laboratories, among children and older adults (age range <1–85 years). Serotypes (O111, O157, O26, O145) and genes (*stx2* only detection and *eaeA* detection) known to cause severe disease (*6*,*7*), were higher for cases diagnosed by other pathology laboratories (Table). Data on subtypes of *stx* were available for 4 SPL and 14 other laboratory cases. *stx2a*, the toxin gene variant reported as being associated with severe disease, was detected only among cases diagnosed by other pathology laboratories (n = 6); all of those cases were symptomatic.

Consistent with current Queensland Health guidelines, all reported STEC cases are investigated and followed up to identify a source of infection (*1*). Case-patients are excluded from working in high-risk settings, and all case-patients, household contacts, and other symptomatic contacts are followed until evidence of microbiological clearance (2 successive negative stool samples 24 hours apart) (*1*). Although asymptomatic case-patients can infect other persons, evidence and guidance for managing asymptomatic cases is varied and less clear (*8*). In low-risk settings, treatment and exclusion of asymptomatic cases might not be necessary (*8*).

*stx* genes can be detected in stool specimens even when bacterial culture is negative (*9*). Use of higher sensitivity PCRs for STEC screening can result in an increase in notifications. A range of STEC virulence factors and host factors can influence clinical manifestations and outcome of infection, and it has been proposed that certain profiles could be useful predictors of strains associated with causing severe illness (*10*). Although causal inference of these factors with severity of disease could not be established, this investigation provided insight into the observation of increasing detection of mild STEC infection and changes in laboratory testing practices, including testing requests by alternative health practitioners.

Management of STEC cases requires resources for follow-up and testing of both symptomatic and asymptomatic case-patients and their contacts. Therefore, reports of asymptomatic cases and changes in testing practices, as shown by this study, suggest a need to revise existing guidelines for the management of STEC cases on the basis of clinical manifestations, laboratory testing, identification of risk-groups, and available resources.

## References

[1] Queensland Health. Shiga toxin-producing *Escherichia coli* (STEC) infection. 2014 Dec 2 [cited 2022 Jul 20]. https://www.health.qld.gov.au/cdcg/index/stec

[2] Morita-Ishihara T, Iyoda S, Iguchi A, Ohnishi M. Secondary Shiga toxin-producing *Escherichia coli* infection, Japan, 2010–2012. Emerg Infect Dis. 2016;22:2181–4. 10.3201/eid2212.160783 27869602 PMC5189154

[3] De Rauw K, Jacobs S, Piérard D. Twenty-seven years of screening for Shiga toxin-producing *Escherichia coli* in a university hospital. Brussels, Belgium, 1987-2014. PLoS One. 2018;13:e0199968. 10.1371/journal.pone.0199968 29965972 PMC6028080

[4] Australian Government Department of Health and Aged Care. Shiga toxin-producing *Escherichia coli* (STEC) infection. 2016 Apr [cited 2022 Jul 31]. https://www.health.gov.au/sites/default/files/documents/2022/06/shiga-toxin-producing-escherichia-coli-stec-infection-surveillance-case-definition.pdf

[5] Australian Government Department of Health and Aged Care. National notifiable disease surveillance system: national communicable disease surveillance dashboard. 2022 [cited 2023 Aug 11]. https://nindss.health.gov.au/pbi-dashboard

[6] UK Health Security Agency. Public health operational guidance for Shiga toxin-producing *Escherichia coli* (STEC). 2023 Jan [cited 2023 Apr 4]. https://assets.publishing.service.gov.uk/government/uploads/system/uploads/attachment_data/file/1127818/health-guidance-shiga-toxin-producing-escherichia-coli.pdf

[7] Persson S, Olsen KE, Ethelberg S, Scheutz F. Subtyping method for *Escherichia coli* shiga toxin (verocytotoxin) 2 variants and correlations to clinical manifestations. J Clin Microbiol. 2007;45:2020–4. 10.1128/JCM.02591-06 17446326 PMC1933035

[8] Shane AL, Mody RK, Crump JA, Tarr PI, Steiner TS, Kotloff K, et al. 2017 Infectious Diseases Society of America clinical practice guidelines for the diagnosis and management of infectious diarrhea. Clin Infect Dis. 2017;65:e45–80. 10.1093/cid/cix669 29053792 PMC5850553

[9] Macori G, McCarthy SC, Burgess CM, Fanning S, Duffy G. Investigation of the causes of shigatoxigenic *Escherichia coli* PCR positive and culture negative samples. Microorganisms. 2020;8:587. 10.3390/microorganisms8040587 32325659 PMC7232186

[10] FAO/WHO STEC EXPERT GROUP. Hazard identification and characterization: criteria for categorizing Shiga toxin-producing *Escherichia coli* on a risk basis. J Food Prot. 2019;82:7–21. 10.4315/0362-028X.JFP-18-291 30586326

